# High-Performance Memristive Synapse Composed of Ferroelectric ZnVO-Based Schottky Junction

**DOI:** 10.3390/nano14060506

**Published:** 2024-03-11

**Authors:** Youngmin Lee, Chulwoong Hong, Sankar Sekar, Sejoon Lee

**Affiliations:** 1 Department of Semiconductor Science, Dongguk University-Seoul, Seoul 04620, Republic of Korea; ymlee@dongguk.edu (Y.L.); ghdcjf0109@dongguk.edu (C.H.); sanssekar@dongguk.edu (S.S.); 2 Quantum-Functional Semiconductor Research Center, Dongguk University-Seoul, Seoul 04620, Republic of Korea

**Keywords:** vanadium-doped ZnO, ferroelectric Schottky junction, synaptic device

## Abstract

In pursuit of realizing neuromorphic computing devices, we demonstrated the high-performance synaptic functions on the top-to-bottom Au/ZnVO/Pt two-terminal ferroelectric Schottky junction (FSJ) device architecture. The active layer of ZnVO exhibited the ferroelectric characteristics because of the broken lattice-translational symmetry, arising from the incorporation of smaller V^5+^ ions into smaller Zn^2+^ host lattice sites. The fabricated FSJ devices displayed an asymmetric hysteresis behavior attributed to the ferroelectric polarization-dependent Schottky field-emission rate difference in between positive and negative bias voltage regions. Additionally, it was observed that the magnitude of the on-state current could be systematically controlled by changing either the amplitude or the width of the applied voltage pulses. Owing to these voltage pulse-tunable multi-state memory characteristics, the device revealed diverse synaptic functions such as short-term memory, dynamic range-tunable long-term memory, and versatile rules in spike time-dependent synaptic plasticity. For the pattern-recognition simulation, furthermore, more than 95% accuracy was recorded when using the optimized experimental device parameters. These findings suggest the ZnVO-based FSJ device holds significant promise for application in next-generation brain-inspired neuromorphic computing systems.

## 1. Introduction

Neuromorphic computing, which is conceived to replicate the highly efficient parallel data computation of the human brain, has emerged as one of the most prospective techniques for realizing future artificial intelligence technology [1,2]. In the field of semiconductor electronics, the core challenge is to emulate the biological synapses, which are the fundamental units of the human brain responsible for information processing (e.g., logic, memory, learning, cognition, etc.). To accomplish this goal, many researchers have devoted themselves to investigating various materials and device architectures that can exhibit versatile synaptic functions with high computational efficiency and low energy consumption. At this point, we need to consider how one can mimic biological synapses to adapt and learn by using electronic devices. When considering the device operation schemes, diverse memory cells can be potential candidates that may implement neuro-inspired arithmetic computation. This is because the pivotal role of the memory cell is the dynamic nature of the biological synapse for storing, modulating, and computing the synaptic weights [3]. Additionally, the controllability of synaptic plasticity is of importance for learning and memory in both biological brains and artificial neural networks. Therefore, memory-based neuromorphic computing systems may offer great potential to achieve efficient brain-mimicked information processes as well as sophisticated cognitive functions. For instance, two-terminal memory devices, such as resistive switching memory [4,5], phase-change memory [6,7], and ferroelectric memory [8,9], and three-terminal devices, such as ferroelectric gate-oxide memory [10,11], charge-trap memory [12,13], and floating-gate memory [14,15], are feasible examples that can demonstrate the analog synaptic memory functions.

Among the various memory device structures, two-terminal ferroelectric memristors may offer exceptional advantages such as simplified device structures, being devoid of extra control terminals, streamlining integration, and having rapid computational speeds [16,17]. These are beneficial for the demonstration of compact and energy-efficient artificial neural networks. For materializing ferroelectric memristor-based synapses, typical ferroelectric materials are often used, such as perovskite ABO_3_ ferroelectric oxides (e.g., BiTiO_3_ [18,19], BiFeO_3_ [20,21], and Pb(Zr,Ti)O_3_ [22,23]), non-perovskite ferroelectric oxides (e.g., HfZrO_2_ [24,25,26,27]), and organic ferroelectric materials (e.g., P(VDF-TeFE) [28,29]). Except for high-quality HfZrO_2_ that can be grown by atomic layer deposition [26,27], however, it is still difficult to grow high-quality thin films of other perovskite materials. Additionally, conventional ferroelectric materials may suffer from sneak current issues because of the strong ferroelectric domain wall motion [30,31]. Therefore, finding an alternative ferroelectric material is essential. Fortunately, there is an exceptional opportunity to use a ferroelectric semiconductor layer because it allows for the growth of high-quality thin films as well as the formation of the ferroelectric Schottky junction (FSJ) structure. In recent years, it has been reported that transition metal-doped ZnO semiconductors could show a ferroelectric nature at elevated temperatures [32,33,34,35]. In particular, vanadium-doped ZnO (ZnVO) represented an innovative approach to replace conventional ferroelectric materials. One of its most striking features is high ferroelectric stability at higher Curie temperatures above 300 K [36,37,38], providing robust solutions to perform the enhanced memory performances such as superior data retention, memory reliability, and explicit switching characteristics. These advantages specify an ample potential of ZnVO as a superior choice for demonstrating robust and reliable synaptic devices. Despite these substantial benefits, ZnVO-based FSJs and their synapse applications have not been investigated.

Motivated by all these backgrounds, we therefore investigated the fabrication and the characterization of high-performance ZnVO-based two-terminal FSJ synaptic devices. The devices were fabricated in the form of top-to-bottom Au/ZnVO/Pt two-terminal FSJ memristors, and exhibited high-performance synaptic functions. Herein, we initially examine the material properties of the ZnVO active layers, such as structural phases, chemical valence states, ferroelectric hysteresis, and electrical switching characteristics, and then we thoroughly assess and discuss the synaptic functions of the Au/ZnVO/Pt FSJ synaptic devices.

## 2. Materials and Methods

**Figure 1:**
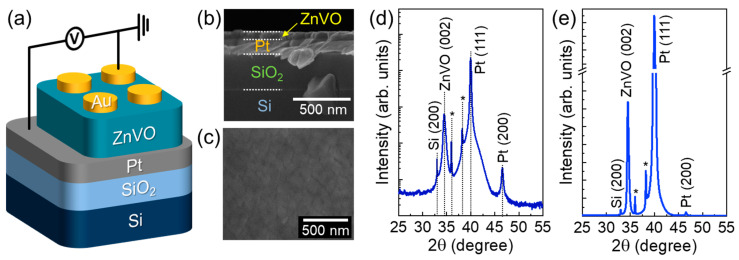
(**a**) Schematic of the Au/ZnVO/Pt FSJ device. (**b**) Cross-sectional FE-SEM image of the ZnVO layer grown on the (111) Pt/SiO_2_/Si substrate. (**c**) Surface FE-SEM image of the ZnVO layer. (**d**) XRD patterns of the ZnVO layer represented in the log scale. (**e**) Linear-scale XRD graph. Small peaks depicted by asterisk marks arose from the Ti adhesion layer between Pt and SiO_2_.

Figure 1a illustrates the schematic of the fabricated memristive FSJ synapse with the top-to-bottom Au/ZnVO/Pt two-terminal device structure. To construct such a device scheme, firstly, we deposited the high-quality (111) Pt layer onto the SiO_2_/Si substrate. Here, we note that, prior to (111) Pt deposition, the ultrathin Ti adhesion layer (~3 nm) was sputter-deposited onto the substrate. Then, the mirror-like (111) Pt layer (~120 nm) was subsequently deposited at 500 °C by D.C. magnetron sputtering using a high-purity Pt (99.999%) target [32]. The base pressure was approximately 10^−7^ Torr, and the working pressure was maintained at 30 mTorr under the Ar (99.9999%, 25 sccm) gas flow state. During the Pt deposition, the D.C. power was 254 W (V ≈ 300 V and I ≈ 0.82 A). Next, we grew the 70 nm thick smooth ZnVO (V: 2.5 at.%) layer onto the (111) Pt/SiO_2_/Si substrate at 200–500 °C by R.F. magnetron sputtering using a 99.99%-purity ZnVO (V: 4.0 wt.%) ceramic target. When growing the ZnVO layer, the R.F. power was 120 W and the working pressure was fixed at 25 mTorr in a gas mixture medium with Ar (99.9999%, 15 sccm) and O_2_ (99.999%, 10–20 sccm). Both D.C. and R.F. magnetron sputtering processes were conducted by using a KVS-2000 system (Korea Vacuum Tech., Gyeonggi, Korea). Soon after the ZnVO growth, the sample was annealed at 550 °C for 60 s in O_2_ to improve its crystal quality. Finally, the 100 μm diametral Au electrodes (*t*_Au_ ≈ 150 nm) were lithographically formed on top of the ZnVO surface.

The surface morphology of ZnVO was scoped by field-emission scanning electron microscopy (FE-SEM, Hitachi S4160, Tokyo, Japan), and the crystallographic structure of ZnVO was monitored by X-ray diffraction (XRD, Rigaku Miniflex 300, Tokyo, Japan). The chemical bonding states were characterized by X-ray photoelectron spectroscopy (XPS, Thermo Fisher Scientific ESCALab250Xi, Waltham, MA, USA). Additionally, we examined the ferroelectric polarization properties of ZnVO by using a Precision RT66C system (Radiant Tech. Inc., Albuquerque, NM, USA). The electrical and synaptic characteristics of the Au/ZnVO/Pt FSJ memristive devices were examined by using a B1500A/B1530A semiconductor device analyzer (Keysight Tech. Inc., Santa Rasa, CA, USA).

## 3. Results and Discussion

In vertically stacked FSJ devices such as our Au/ZnVO/Pt scheme, the morphological surface texture is of importance because the microstructural faults, such as such grain boundaries, hillocks, and pinholes, may create a leakage path inside the vertical FSJ device. This will, in turn, eventually degrade the ferroelectric switching and electrical transport characteristics of the FSJ device. Thus, firstly, we devoted ourselves to obtaining smooth and well-merged ZnVO layers by controlling both the growth temperature and the oxygen partial pressure. When changing the substrate temperature from 200 to 500 °C (100 °C step), the 300 °C-grown ZnVO layer exhibited a smooth surface texture with no grain agglomeration (Figure S1a–d). Additionally, when varying the oxygen flow rate from 0 to 20 sccm (5 sccm step), it was found that the formation of hillocks and pinholes was drastically diminished at the gas flow condition of ‘Ar:O_2_ = 10 sccm:20 sccm’ (Figure S2a–d). Based on these results, we could grow the 70 nm thick high-quality ZnVO layer at 300 °C in gas ambiance with Ar (10 sccm) and O_2_ (20 sccm) (Figure 1b,c, see also Figure S3). From the XRD analysis, the sample revealed the clear diffraction pattern of the wurtzite (002) ZnVO phase (Figure 1d), and exhibited only a small broadening of the (002) phase (Figure 1e). Furthermore, no secondary phases from V-related precipitates were observed, except for two negligible peaks from the Ti adhesion layer. These depict that the high-quality (002) ZnVO layer was homogeneously grown onto the Pt metal layer.

**Figure 2:**
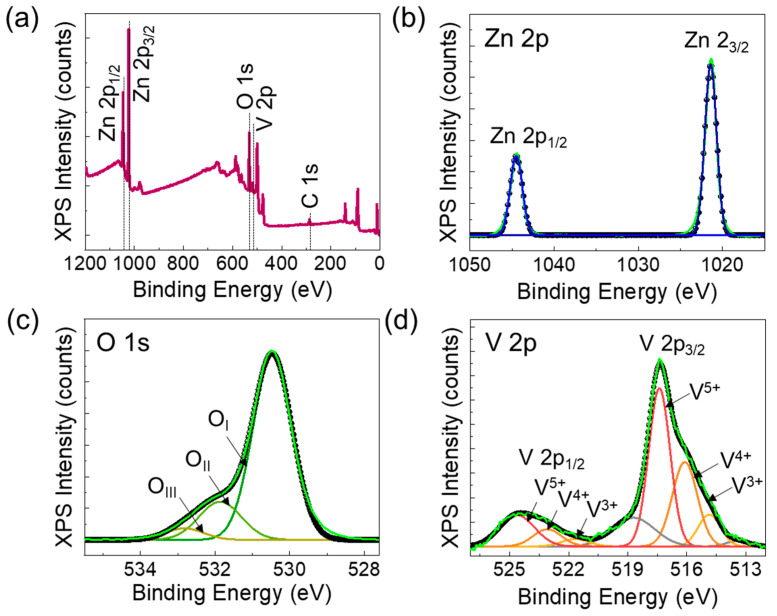
XPS spectra measured from the ZnVO layer: (**a**) survey scan, (**b**) Zn 2p, (**c**) O 1s, and (**d**) V 2p core levels.

Next, we examined the elemental compositions and the chemical bonding states of the ZnVO layer. As can be seen from Figure 2a, the XPS survey spectrum clearly displayed that the ZnVO layer involves only its intrinsic species of Zn, V, and O. From the XPS quantitative analysis, we confirmed that 2.5 at.% of V contents were incorporated in the ZnVO layer. We here note that 2.5 at.% of V was chosen on the basis of the following reasons. According to a previous report [39], the crystal phase segregation might occur when V contents exceeded 4 at.%. Additionally, it was reported that the lattice displacement in ZnVO could be optimized when 2–3 at.% of V was incorporated into the host material–ZnO [40]. The small C 1s peak in the survey spectrum is known to arise from adventitious hydrocarbon that is inevitably detected in the XPS chamber [41]. Thus, the peak position of C 1s (284.6 eV) is typically utilized as a reference binding-energy value for calibrating the core level positions of other species [42]. In the high-resolution Zn 2p spectrum (Figure 2b), two predominant Zn 2p_1/2_ and Zn 2p_3/2_ peaks appeared at 1021.4 and 1044.5 eV, respectively. The binding energy gap between the two peaks was 23.1 eV, corresponding to that of the +2 valence state for the Zn species [43,44]. In the case of O 1s (Figure 2c), the asymmetric feature was observed, and the spectrum could be deconvoluted into the three different components. Namely, the surface-residing loosely bound oxygen ions (~533.0 eV), oxygen vacancies (~531.9 eV), and covalently bonded O^2-^ ions (~530.5 eV) were included in the ZnVO layer [45,46]. Figure 2d shows the bonding states of V, which are of the most significance in ZnVO because the valence states of V ions are directly related to the ferroelectric nature of ZnVO. As depicted in Figure 2d, the V 2p spectrum could be deconvoluted into the three different ionic constituents. Namely, the spin–orbit doublet of both V 2p_1/2_ and V 2p_3/2_ could be primarily fitted to the three valence states: (i) V^5+^ (i.e., 524.63 eV for V 2p_1/2_ and 517.38 eV for V 2p_3/2_), (ii) V^4+^ (523.09 eV for V 2p_1/2_ and 516.20 eV for V 2p_3/2_), and (iii) V^3+^ (521.67 eV for V 2p_1/2_ and 515.19 eV for V 2p_3/2_) [47]. The gray curve centered at 518.7 eV corresponds to the satellite of the O 1s core level [48]. As can be confirmed from the deconvoluted spectra, the portion of V^5+^ is predominant in the present ZnVO layer. Hence, it can be inferred that the penta-valent V ions were effectively incorporated into ZnVO. In other words, the positively ionized V^5+^ (ionic radius: 0.54 Å [47]) dopants were well substituted to the Zn^2+^ (ionic radius: 0.74 Å [32]) cation sites in the ZnO host lattices.

**Figure 3:**
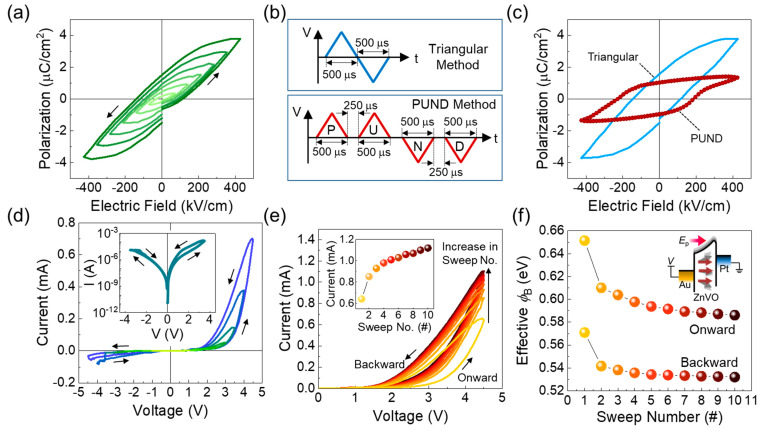
(**a**) P–E characteristic curves of ZnVO measured under various electric-field sweeping ranges. (**b**) Applied pulse schemes for the typical triangular mode and the PUND mode. (**c**) P–E characteristic curves of ZnVO measured by the typical triangular method and the PUND method. (**d**) I–V characteristic curves of the Au/ZnVO/Pt FSJ device measured under various *V*_sweep_ ranges. The inset shows the I–V curve at a semi-logarithmic scale. (**e**) I–V characteristic curves at the positive bias voltage region measured under 10 rounds of consecutive voltage sweeping with a *V*_sweep_ of 4.5 V. The inset represents the variation in the maximum currents as a function of the sweeping number. (**f**) Effective *ϕ*_B_ for both onward and backward sweeps as a function of the sweeping number. The inset represents the gradual decrease in *ϕ*_B_ upon increasing the sweeping number.

In transition metal-doped ZnO, the smaller dopant ions residing at the bigger Zn^2+^ sites significantly give rise to the increase in the local lattice vibrations [49]. In addition, both theoretical [50,51] and experimental studies [52,53,54,55,56] suggested and substantiated that the lattice displacement led to the ferroelectric nature in transition metal-doped ZnO. Therefore, we evaluated the ferroelectric properties of the prepared ZnVO layer. As shown in the polarization vs. electric field (P–E) characteristic curves (Figure 3a), the Au/ZnVO/Pt sample clearly exhibited ferroelectric hysteresis loops, in which both the coercive field (*E*_c_) and the remnant polarization (*P*_r_) were increased with increasing magnitude of the electric-field sweeping range (*E*_sweep_). When *E*_sweep_ = |±430 kV/cm|, *P*_r_ and *E*_c_ were 1.51 μC/cm^2^ and 168 kV/cm, respectively. From multiple samples that were devised by identical process conditions, a similar feature was observed (Figure S4).

For more clarity on the ferroelectric nature of ZnVO, we assessed the ferroelectric-switching response characteristics by utilizing the positive-up–negative-down (PUND) method, which can eliminate the impact of movable defects or non-ferroelectric components. Namely, when using the P, U, N, and D pulses with the appropriate pulse-to-pulse interval (Figure 3b), the polarization charge with only switching components could be extracted by subtracting the U- and D-stimulated response signals from the P- and N-induced response signals, respectively (i.e., polarization charge = [(P − U) + (N − D)]/2) [57,58]. Here, the pulse-to-pulse interval plays a key role in distinguishing both switching and non-switching components before applying the P–U–N–D pulses. As shown in Figure 3c, the PUND curve clearly revealed the typical shape of the ferroelectric hysteresis loop. From the PUND curve, *P*_r_ and *E*_c_ of ZnVO were confirmed to be 1.06 μC/cm^2^ and 230 kV/cm, respectively.

As aforementioned, such a clear ferroelectric characteristic could be attributed to the polar crystalline symmetry, arising from the ionic radii difference between V^5+^ (0.54 Å) and Zn^2+^ (0.74 Å). Namely, the smaller V^5+^ ions would occupy the off-centered positions when they are substituted into the tetrahedrally coordinated host lattice sites of the bigger Zn^2+^ ions [49,54]. This could eventually create localized dipoles within the ZnVO lattices; hence, the ferroelectric nature would exist in the whole ZnVO solid-state system. We therefore ascribe the ferroelectric behavior in our ZnVO to its broken lattice-translational symmetry, originating from the incorporation of smaller V^5+^ ions into bigger Zn^2+^ sites.

After confirming the ferroelectric properties of ZnVO, we evaluated its ferroelectric polarization-dependent resistive switching characteristics. As can be seen from the current vs. voltage (I–V) characteristic curves (Figure 3d), the Au/ZnVO/Pt FSJ device exhibited asymmetric hysteresis loops in negative and positive voltage regions. Namely, the clear hysteresis loops and the reasonable on-state current values appeared in the positive voltage region, while only negligible loops with minimal current values appeared in the negative voltage region (see also the inset of Figure 3d). Additionally, the hysteresis loops in the positive bias voltage region became larger with increasing magnitude of the sweep voltage range (*V*_sweep_). We here note that more than 90% of the devices, fabricated under the same experimental conditions, exhibited a similar asymmetric hysteresis feature (Figure S5). Such a voltage polarity-dependent asymmetric hysteresis behavior can offer several advantages over the typical symmetric hysteresis characteristics (e.g., butterfly shape of symmetric hysteresis loops in negative and positive voltage regions [59,60,61]). For example, different from the symmetric hysteresis case, the asymmetric hysteresis characteristics with a rectifying behavior might allow explicit program/erase operations by switching only the applied voltage polarity because there is only a minimal current flow during the erase operation [62]. Furthermore, the explicit on/off switching characteristics could effectively release the sneak current issue in the cross-bar array of two-terminal synapses’ networks [63].

To help understand the charge conduction and the switching mechanisms, we fitted the I–V curves to the well-known space charge-limited conduction model [64] and the Schottky emission model [64] (see Figure S6a–d). The I–V curve in the lower voltage region below ~1 V was well fitted to the space charge-limited conduction model, while the I–V curve in the higher voltage region above ~1 V was well fitted to the Schottky emission model. Namely, in the lower voltage region, the memristive switching behavior could initially start with the space charge-limited conduction via charge trapping and detrapping at the defect sites. After fully filling the trap sites in the higher voltage region, the charge transport mechanism would, in turn, be changed into the Schottky emission. Thus, it can be conjectured that memristive switching in the on-state current regime is mostly governed by ferroelectric polarization switching.

Here, it should be noticeable that the hysteresis characteristics could be modulated by applying consecutive *V*_sweep_. As shown in Figure 3e, when applying consecutive *V*_sweep_ 10 times, interestingly, the current level gradually increased with increasing number of sweeping cycles (see also the inset of Figure 3e). To quantitatively understand the sweeping-cycle-dependent on-state current variation, we determined the effective Schottky barrier height (*ϕ*_B_) by using the well-known Schottky equation [32]:

(1) $$J=J_{0}\exp(qV/\eta kT)$$

(2) $$J_{0}=AA^{*}T^{2}exp(-q\phi_{B}/kT),$$

where *J*_0_ is the reverse saturation current, *q* is the electron charge, *η* is the ideality factor, *k* is the Boltzmann constant, T is the absolute temperature, A is the contact area, and A* is the Richardson constant (32 A·cm^−2^K^−2^ for ZnO [65]). As plotted in Figure 3f, the initial *ϕ*_B_ values were determined to be ~0.65 and ~0.57 eV at the first stage of onward and backward sweeping, respectively, and these are in agreement with the literature values [65]. As the sweep number increased, the *ϕ*_B_ values in the onward and backward sweeping cycles were decreased to ~0.59 and ~0.53 eV, respectively. This can be interpreted by the accumulated ferroelectric dipole moments (*E*_p_) that are continuously created and retained by consecutive *V*_sweep_. Namely, the effective *ϕ*_B_ would gradually decrease with increasing sweep number (Figure 3f, inset), as discussed in detail later.

**Figure 4:**
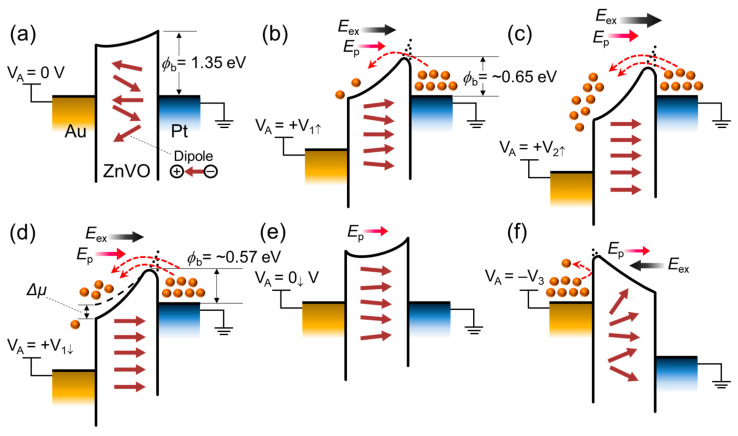
Energy band diagrams of the Au/ZnVO/Pt FSJ device in various bias conditions: (**a**) V_A_ = 0 V, (**b**) V_A_ = +V_1↑_, (**c**) V_A_ = +V_2↑_, (**d**) V_A_ = +V_1↓_, (**e**) V_A_ = 0_↓_V, and (**f**) V_A_ = −V_3_. The dotted lines at the Schottky interfaces in (**b**–**d**,**f**) represent the image force barrier lowering effect.

Prior to discussing the synaptic characteristics of the Au/ZnVO/Pt FSJ device, we here explain the plausible mechanism of the rectified asymmetric hysteresis behavior. Figure 4 illustrates the energy band diagrams of the Au/ZnVO/Pt FSJ device in various bias conditions. At thermal equilibrium (Figure 4a), the Schottky barriers would be created at both Au/ZnVO and ZnVO/Pt interfacial regions because the work function energy values of both Au (Φ_Au_ ≈ 5.1 eV [66]) and Pt (Φ_Pt_ ≈ 5.7 eV [66]) are much greater than the electron affinity of the host material ZnO (χ_ZnO_ ≈ 4.1 eV [65,67,68]). In addition, since Φ_Pt_ > Φ_Au_, the Schottky barrier height on the ZnVO/Pt side should be larger than that on the Au/ZnVO side. Under the Pt-grounded condition, therefore, the Schottky field emission could easily occur on the ZnVO/Pt side when applying the positive upward bias voltage stress (e.g., *V*_A_ = +*V*_1↑_) to the Au electrode terminal (Figure 4b). Namely, because of both the image force barrier lowering effect and the greater electric field on the ZnVO/Pt side (which is greater than that on the Au/ZnVO side), the effective Schottky barrier would easily become low and thin enough to ensure the electron emission from Pt to Au through ZnVO. At the same time, the external electric field (*E*_ex_) from +*V*_1↑_ would lead to the dipole alignment inside the ferroelectric ZnVO layer. Thus, the additional field from the ferroelectric dipole moment (*E*_p_) would be created along with the *E*_ex_ direction.

As one increased the applied upward voltage (e.g., *V*_A_ = +*V*_2↑_), the electron emission probability would become larger than that at +*V*_1↑_ (Figure 4c). When assuming that the magnitude of +*V*_2↑_ exceeds the coercive voltage (+*V*_c_) of ZnVO, the ZnVO layer should be laid on the full polarization state with the non-volatile *E*_p_. When decreasing the applied downward voltage back to +*V*_1↓_ (Figure 4d), therefore, the electron emission probability would remain high because the ferroelectrically retained *E*_p_ still holds the potential gradient inside the ferroelectric ZnVO layer. In other words, the potential difference (Δ*μ*) could occur in the two bias conditions between upward +*V*_1↑_ and downward +*V*_1↓_ even though the voltage magnitudes are same in both +*V*_1↑_ and +*V*_1↓_. This could eventually lead to a larger electron emission rate at +*V*_1↓_ than at +*V*_1↑_, and it could result in the hysteresis behavior in the I–V characteristic curve. Such a retainable *E*_p_ component would not be smeared out unless the ferroelectric dipoles are unpolarized at −*V*_c_. When returning back to zero bias (e.g., *V*_A_ = 0_↓_ V), thus, the *E*_p_ should retain inside the ZnVO layer, while the electron emission should be drastically reduced because of the increased effective Schottky barrier width (Figure 4e). Under this circumstance, the applied negative voltage (e.g., *V*_A_ = −*V*_3_) would be primarily spent on depoling the ferroelectric dipoles. Namely, the effective Schottky barrier would remain high and thick on the Au/ZnVO side because the large portion of −*V*_3_ should compensate the *E*_p_ that was created by +*V*_2↑_. This behavior will be maintained unless the magnitude of |−*V*_A_| largely exceeds |−*V*_c_|. At a moderate |−*V*_A_| below |−*V*_c_|, therefore, the Schottky emission rate would be kept low so that no hysteretic behavior occurs in the −*V*_A↑↓_ region (Figure 4f). When repeating these switching cycles, the effective *ϕ*_B_ value would gradually decrease because of the retained *E*_p_ and its corresponding increase in *Δμ.* This could eventually lead to a gradual increase in the Schottky emission rate so that the on-state current would gradually increase upon increasing switching cycles (e.g., increasing sweep numbers or pulse numbers).

**Figure 5:**
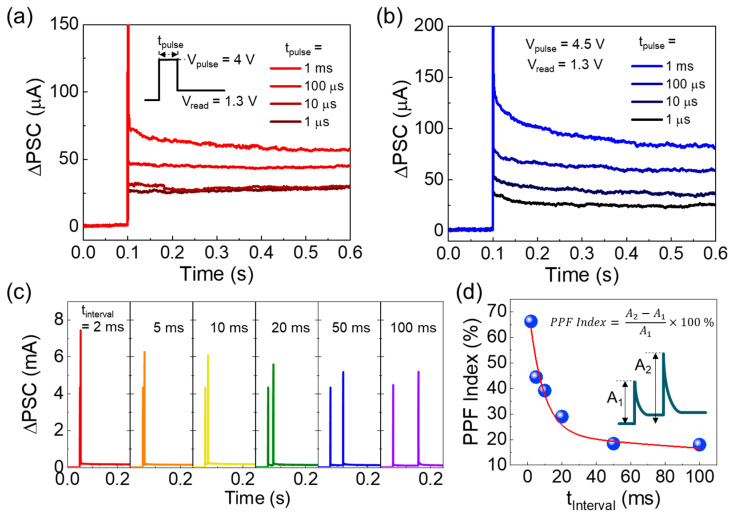
Basic synaptic characteristics of Au/ZnVO/Pt FSJ device: (**a**) EPSC functions performed by applying *V*_pulse_ of 4 V with *t*_pulse_ of 1 μs–1 ms, (**b**) EPSC functions performed by applying *V*_pulse_ of 4.5 V with *t*_pulse_ of 1 μs–1 ms, (**c**) dependence of PPF characteristics on *t*_interval_ (here, *V*_pulse_ and *t*_pulse_ were fixed at 4.5 V and 1 ms, respectively), and (**d**) PPF index as function of *t*_interval_.

The ferroelectric *E*_p_-dependent *ϕ*_B_ variation and its corresponding memristive characteristics could offer a significant advantage for demonstrating synaptic functions. Based upon this feature, we emulated the biological synaptic functions by using the present Au/ZnVO/Pt FSJ device. Figure 5a,b show the transient characteristics of excitatory postsynaptic current (EPSC) after applying a single presynaptic stimulus with pulse amplitudes (*V*_pulse_) of 4 and 4.5 V, respectively. Here, the read-out voltage (*V*_read_) was fixed constant at 1.3 V, while the pulse width (*t*_pulse_) was varied from 1 μs to 1 ms. In both cases (Figure 5a,b), the device showed the typical EPSC transient curves after applying the single pulse stimulus. Namely, the electric pulse-stimulated postsynaptic current (ΔPSC) was rapidly stabilized after its initial decay (see also Figure S7a). In addition, the magnitude of residual ΔPSC increased with increasing *t*_pulse_. Notably, the device revealed a *t*_pulse_-dependent gradual ΔPSC augmentation, particularly when applying the 4.5 V pulse stimulus. Furthermore, the residual ΔPSC value for each t_pulse_ was greater when *V*_pulse_ was 4.5 V compared to that when *V*_pulse_ was 4.0 V. These behaviors are quite similar to those of the biological synapse. In other words, the synaptic plasticity in biological systems depends on both the strength and the duration of incident stimuli. Thus, it can be conjectured that the infusion of the relatively modest stimuli may strengthen the synaptic plasticity so that our Au/ZnVO/Pt FSJ device can mimic the biological synapse.

To verify the above hypothesis, we examined the short-term memory (STM) and the long-term memory (LTM) characteristics of the Au/ZnVO/Pt FSJ device. Firstly, the paired pulse facilitation (PPF) characteristics were assessed to evaluate how effectively the device can perform short-term synaptic strength. PPF quantifies the amplification ratio of ΔPSC in between the first and the second peaks, corresponding to the first and the second pulse stimuli, respectively. At this point, the pulse-to-pulse interval (*t*_interval_) between the two pulses is of importance because the second stimulus could primarily contribute to updating the short-term synaptic strength. Thus, we measured the variation in ΔPSC with respect to *t*_interval_. Similar to EPSC, the PPF curves also showed typical transient responses to the applied pulses (see Figure S7b). In the PPF case, however, the second ΔPSC value increased after applying the second pulse from the paired pulses (Figure 5c). Here, it should be noticed that the magnitude of ΔPSC decreased with increasing *t*_interval_. This is because, during long *t*_interval_, the ferroelectrically polarized dipoles were somewhat depoled so that their memory retention weakened. Additionally, the discrepancy between the two peaks (i.e., *A*_2_ − *A*_1_, see the inset of Figure 5d) tended to decrease with increasing *t*_interval_ (Figure 5d). Accordingly, the PPF index (i.e., (*A*_2_ − *A*_1_)/*A*_1_ × 100%) exponentially decayed with increasing *t*_interval_. Such a PPF decay can be attributable to two distinct relaxation phases (i.e., rapid and slow) [69,70]:

(3) $$PPF\;index=C_{1}exp\left(-t_{interval}/\tau_{1}\right)+C_{2}exp\left(-t_{interval}/\tau_{2}\right),$$

where *τ*_1_ and *τ*_2_ are the time constants for the rapid and slow relaxation phases, respectively, and *C*_1_ and *C*_2_ denote the initially facilitated values for the rapid and slow relaxation phases, respectively. From the fitting curve (see the red line in Figure 5d), *τ*_1_ and *τ*_2_ were determined to be 8.34 and 352.09 ms, respectively, and these values belong to the reasonable range of the biological synapse [71]. This specifies that the present Au/ZnVO/Pt FSJ device could splendidly replicate the biological synapse.

**Figure 6:**
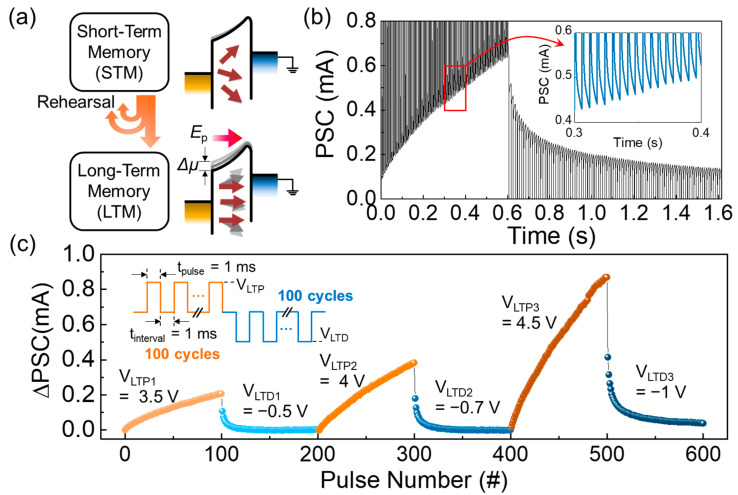
(**a**) Conceptual illustrations of the conversion of the synaptic state from STM to LTM. (**b**) LTP and LTD characteristics of the Au/ZnVO/Pt FSJ device measured by applying 100 consecutive LTP pulses (*V*_pulse_ = 4 V, *t*_pulse_ = 1 ms, and *t*_interval_ = 5 ms) and 100 consecutive LTD pulses (*V*_pulse_ = −1 V, *t*_pulse_ = 100 μs, and *t*_interval_ = 10 ms), respectively. (**c**) Dependences of the ΔPCS dynamic ranges on the magnitudes of *V*_LTP_ and *V*_LTP_ (here, *t*_pulse_ and *t*_interval_ were fixed at 1 ms and 1 ms, respectively).

In the biological synapse, the transfer of selected information from STM to LTM represents a fundamental synaptic learning rule. LTM signifies the permanent change in synaptic weights, updating from the high-frequency consecutive stimuli, while STM rapidly reverts to the initial state from the temporarily updated memory state. Thus, similar to the rehearsal process in the human brain [69,72], repetitive practice may enhance the transition probability from STM to LTM (see the left-hand-side panel in Figure 6a). In our device scheme, such a rehearsal process can be demonstrated by gradual thinning of the Schottky barrier width. As discussed above, the non-volatile *E*_p_, created by the applied electrical pulse, yields a change in Δ*μ* (Figure 4d). Additionally, it was also observed that the magnitude of ΔPSC depends on the pulse parameters of *V*_pulse_, *t*_pulse_, and *t*_interval_ (Figure 5a–d). These infer that, when choosing the appropriate pulse parameters, the magnitude of Δ*μ* can be gradually increased by applying the consecutive pulses because the degree of polarization can also be gradually increased by applying the consecutive pulses (see the right-hand-side panel in Figure 6a). Hence, the effective Schottky barrier width would become thinner and thinner upon increasing the number of pulse stimuli, resulting in enhanced retention with increased conductivity. Based on this idea, we examined the LTM characteristics of the Au/ZnVO/Pt FSJ device. As shown in Figure 6b, the device clearly exhibited long-term potentiation (LTP) and long-term depression (LTD) characteristics when the device was subjected to 100 consecutive LTP pulses (*V*_pulse_ = 4 V, *t*_pulse_ = 1 ms, and *t*_interval_ = 5 ms) and 100 consecutive LTD pulses (*V*_pulse_ = −1 V, *t*_pulse_ = 100 μs, and *t*_interval_ = 10 ms). The sequentially updated synaptic weights can also be traced from the inset of Figure 6b.

After observing the clear LTP/LTD functions, we evaluated the dependence of the ΔPSC dynamic range on the *V*_pulse_ magnitude because the ΔPSC value relies on the variation of Δ*μ*, which is predominantly dependent on *E*_p_ (∝ *V*_pulse_). For this assessment, we only varied the pulse magnitudes for both LTP and LTD (i.e., *V*_LTP_ and *V*_LTP_), while *t*_pulse_, *t*_interval_, and *V*_read_ were fixed at 1 ms, 1 ms, and 1.3 V, respectively. As a result, the dynamic range of ΔPSC increased with increasing *V*_LTP_ and *V*_LTP_ (Figure 6c). From the application point of view, the electronic synapse should possess not only a wide dynamic range but also a good linearity because both of the two factors are essential to improve the learning accuracy as well as the training efficiency of the synapse. The linearity of the synapse can be quantitatively analyzed by following equation [73]:

(4) $$G_{P}=G_{min}+G_{0}\left(1-e^{-Ap}\right)$$

(5) $$G_{D}=G_{max}-G_{0}\left(1-e^{A\left(p-1\right)}\right)$$

(6) $$G_{0}=\frac{G_{max}-G_{min}}{1-e^{-Ap}},$$

where *G*_P_ and *G*_D_ denote the conductance values for LTP and LTD, respectively; *G*_min_ and *G*_max_ are the minimum and the maximum conductance values, respectively; and *p* is the number of applied pulses. *A* is the fitting parameter that presents the nonlinearity of the synaptic weights with respect to the applied pulses. For instance, |*A*| becomes large when the LTP/LTD curves are convex, whereas |*A*| converges to zero when the LTP/LTD curves are linear. By fitting the experimental LTP and LTM data to the above equations, the |*A*| values were calculated to be 0.91–1.03 for LTP and 7.05–9.25 for LTD. The high degree of nonlinearity in the LTD mode is thought to result from the early depression (i.e., abrupt change in ΔPSC at the initial LTD pulse stage), presumably because of the small reverse saturation current in the negative bias voltage region (see Figure 3b). To improve the linearity in the LTD mode, improving the rectification characteristics can be the next step for realizing future high-performance neuromorphic synapse networks (e.g., reducing the oxygen vacancies inside the ferroelectric layer and/or decreasing the interfacial states at the metal/ferroelectric junction).

**Figure 7:**
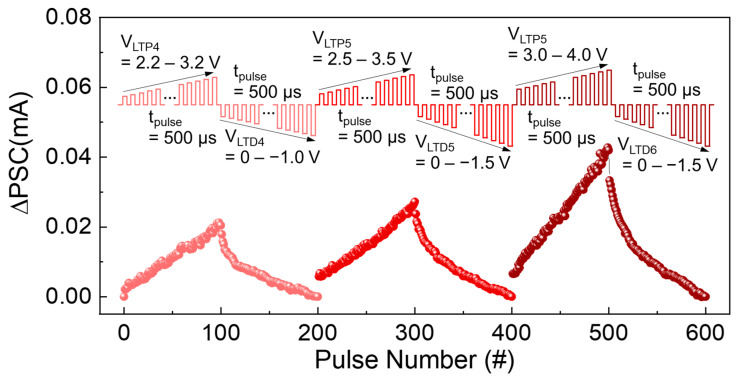
LTP and LTD characteristics of the Au/ZnVO/Pt FSJ device measured under the incremental *V*_pulse_ scheme. The inset illustrates the linearly increased *V*_LTP_ and *V*_LTD_ pulse schemes performed for this measurement.

Although the above issue (i.e., improving the LTD linearity) is still challenging in two-terminal synaptic devices [74,75], utilizing the pulse modulation methods (e.g., using the incremental pulse scheme [76,77] and/or changing the pulse frequency [78,79]) can be an effective solution to improve both the linearity and the symmetricity for reliable LTP/LTD functions. We therefore tried to find the proper pulse schemes by modulating the magnitude of *V*_pulse_. As represented in the inset of Figure 7, we chose incremental pulse schemes for both *V*_LTP_ and *V*_LTP_, while *t*_pulse_, *t*_interval_, and *V*_read_ were fixed at 500 μs, 500 μs, and 1.3 V, respectively. Compared to the case of the identical pulse scheme (Figure 6c), the linearity was significantly improved when using the incremental pulse scheme (Figure 7). Through fitting the experimental date to Equations (2)–(4), we confirmed that the |*A*| values were improved to 0.34–0.72 for LTP and 2.13–2.17 for LTD when the incremental pulse schemes were subjected to the device.

**Figure 8:**
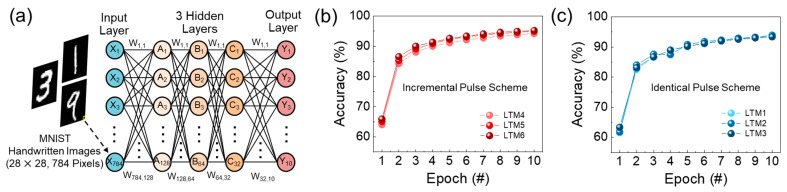
(**a**) Schematic illustration of the artificial neural network used for the MNIST simulation. (**b**) Simulated pattern recognition accuracy when using the experimental LTP/LTD data obtained from the case of the identical pulse scheme (Figure 6c). (**c**) Simulated pattern recognition accuracy for the case of the incremental pulse scheme (Figure 7).

As aforementioned, the improved linearity is closely related to the deep learning accuracy of the synapse. We therefore tested the pattern recognition accuracy within an artificial neural network system, which is based upon the backpropagation learning rule in a neuromorphic system. For this test, we employed a synthetic multilayer neural network, comprising one input layer, three hidden layers, and one output layer (Figure 8a). Here, we note that the pattern recognition accuracy was assessed by theoretical simulation using the Modified National Institute of Standard and Technology (MNIST) handwritten digit dataset, in which 60,000 handwritten training images and 10,000 testing images are included. Each handwritten training image involves 28 × 28 pixels, and they are converted into the 784 input vector neurons at the input layer. Those vectors are delivered to the 10 output neurons via propagating through the 128 → 64 → 32 nodes of the hidden layers. Then, the output layer calculates the recognition accuracy by comparing the updated synaptic weights and the database values. Through multiple simulation runs using the experimental parameters from Figure 6c and Figure 7, we found that the recognition accuracy was improved when using the incremental pulse scheme. For example, after 10 epochs, the pattern recognition accuracy reached 95.1–95.3% for the incremental pulse scheme (Figure 8b), whereas this was 93.4–93.9% for the identical pulse scheme (Figure 8c).

**Figure 9:**
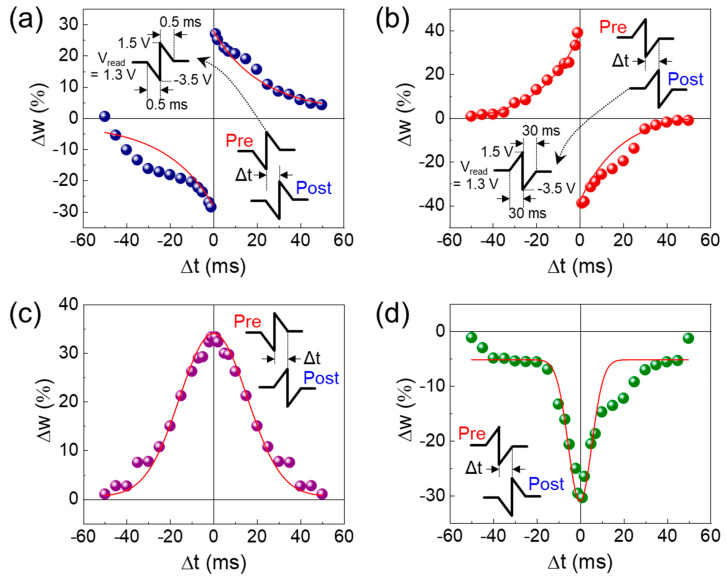
Versatile STDP characteristics of the Au/ZnVO/Pt FSJ device: (**a**) asymmetric Hebbian rule, (**b**) asymmetric anti-Hebbian rule, (**c**) symmetric Hebbian rule, and (**d**) symmetric anti-Hebbian rule. Each inset illustrates the used spike pulse scheme for demonstrating each Hebbian rule. The red line in each figure depicts the fitting curve.

Finally, we examined the spike-timing-dependent plasticity (STDP) characteristics, which are of importance for emulating the perceptron role of the synapse in the neural network [80,81,82]. In electronic synapses, STDP is typically characterized by measuring the chance in synaptic weights (Δ*w*), depending on the time difference between the simultaneously applied pulse pair of the presynaptic and the postsynaptic stimuli (i.e., Δ*t* = *t*_post_ − *t*_pre_). Thus, STDP can allow us to assess the synaptic perceptron role that discriminates the temporal difference in synaptic states between the presynapse and the postsynapse. As shown in Figure 9, by changing the polarities of the applied pair pulses (see each inset of the figure), we efficaciously demonstrate the four different Hebbian STDP learning rules on our Au/ZnVO/Pt FSJ device, i.e., (i) the asymmetric Hebbian rule (Figure 9a), (ii) the asymmetric anti-Hebbian rule (Figure 9b), (iii) the symmetric Hebbian rule (Figure 9c), and (iv) the symmetric anti-Hebbian rule (Figure 9d). In all cases, Δ*w* tended to decay as Δ*t* was increased. Such a Δ*t*-dependent Δ*w* decay characteristic can be further analyzed by parametrizing the STDP time constant (*τ*_s_) by using the following equations [83]:

(7) $$∆w=Aexp\left(-\frac{\Delta t}{\tau_{s}}\right)+∆w_{0}\;(\mathrm{for}\;\mathrm{asymmetric}\;\mathrm{Hebbian}\;\mathrm{rules})$$

(8) $$∆w=Aexp\left(-\frac{\Delta t^{2}}{\tau_{s}^{2}}\right)+∆w_{0}\;(\mathrm{for}\;\mathrm{symmetric}\;\mathrm{Hebbian}\;\mathrm{rules}),$$

where *A* is the scaling factor and Δ*w*_0_ is the constant value that is non-associative to the synaptic weight change. By fitting the experimental parameters to the above equations, the *τ*_s_ values were extracted to be (i) 22.58, (ii) 18.84, (iii) 21.99, and (iv) 7.63 ms from Figure 9a–d, respectively. The obtained *τ*_s_ values, i.e., timescales of a few tens of milliseconds, are analogous to those of the biological synapses in the human brain [84]. Furthermore, the rapid change in Δ*w* can be a substantial benefit for the neural network circuit design because the temporal Δ*w* change within a peripheral Δ*t* range (i.e., short *τ*_s_) is of excellent use for parallel computing in future neuromorphic computing systems.

## 4. Conclusions

The high-performance synaptic functions were magnificently demonstrated on the ZnVO-based memristive FSJ device scheme. The ZnVO active layer displayed clear ferroelectric hysteresis characteristics attributed to the polar crystalline symmetry due to the incorporation of smaller V^5+^ ions in bigger Zn^2+^ sites. Because of the non-volatile *E*_p_ nature in ZnVO, the FSJ device exhibited voltage polarity-rectifiable asymmetric hysteresis behavior in its I–V characteristics. Additionally, the *E*_p_-adjustable Schottky field-emission rate allowed us to effectively represent both voltage pulse amplitude- and width-tunable multiple memory states. Using these astonishing characteristics, diverse synaptic functions such as EPSC, PPF, LTM, and STDP were efficaciously demonstrated. Through MNIST pattern recognition simulations, the device was shown to achieve a high accuracy up to 95.3%. Furthermore, it was also observed that the device could accomplish the versatile STDP Hebbian learning rules within a timescale of a few tens of ms. These findings suggest that the ZnVO-based FSJ device holds significant promise for next-generation brain-inspired neuromorphic computing systems.

## Data Availability

Data are contained within the article and Supplementary Materials.

## Supplementary Materials

The following supporting information can be downloaded at: https://www.mdpi.com/article/10.3390/nano14060506/s1, Figure S1: Surface FE-SEM image of the ZnVO layers grown at different temperatures: (a) 200 °C, (b) 300 °C, (c) 400 °C, and (d) 500 °C; Figure S2: Surface FE-SEM image of the ZnVO layers grown under different gas flow conditions: (a) Ar:O_2_ = 15 sccm:10 sccm, (b) Ar:O_2_ = 15 sccm:15 sccm, (c) Ar:O_2_ = 15 sccm:18 sccm, and (d) Ar:O_2_ = 15 sccm:20 sccm; Figure S3: Surface AFM image of the ZnVO layer grown at 300 °C in gas ambiance with Ar (10 sccm) and O_2_ (20 sccm); Figure S4: (a–d) P–E characteristic curves of the multiple ZnVO samples (obtained from different growth batches) grown at 300 °C in a gas mixture of Ar (10 sccm) and O_2_ (20 sccm); Figure S5: (a–d) I–V characteristic curves of the multiple Au/ZnVO/Pt FSJ devices that were fabricated using different ZnVO layers obtained from different growth batches. For all cases, the growth temperature and the gas flow rate were fixed at 300 °C and Ar/O_2_ = 10 sccm:20 sccm, respectively; Figure S6: I–V characteristic curves of the Au/ZnVO/Pt FSJ device represented in (a) semi-logarithmic scale, (b) log–log scale, (c) space charge-limited conduction plot, and (d) Schottky plot; Figure S7: Transient characteristic curves for (a) EPSC and (d) PPF of the Au/ZnVO/Pt FSJ device.
