# Supplementary material for: High-Performance Memristive Synapse Composed of Ferroelectric ZnVO-Based Schottky Junction
Source: Nanomaterials (Basel). 2024 Mar 11;14(6):506. doi: 10.3390/nano14060506 (PMC10974313; doi:10.3390/nano14060506)
Supplement: Supplementary file 1 [file nanomaterials-14-00506-s001.zip › nanomaterials-2860456-supplementary.pdf]

nanomaterials

Supplementary Materials

High-Performance Memristive Synapse Composed of Ferroelectric ZnVO-Based Schottky Junction

Youngmin Lee [1,2], Chul-Woong Hong [1], Sankar Sekar [1,2], and Sejoon Lee [1,2,*]

[1] Department of Semiconductor Science, Dongguk University-Seoul, Seoul 04620, Republic of Korea
[2] Quantum-functional Semiconductor Research Center, Dongguk University-Seoul, Seoul 04620, Republic of Korea
* Correspondence: sejoon@dongguk.edu

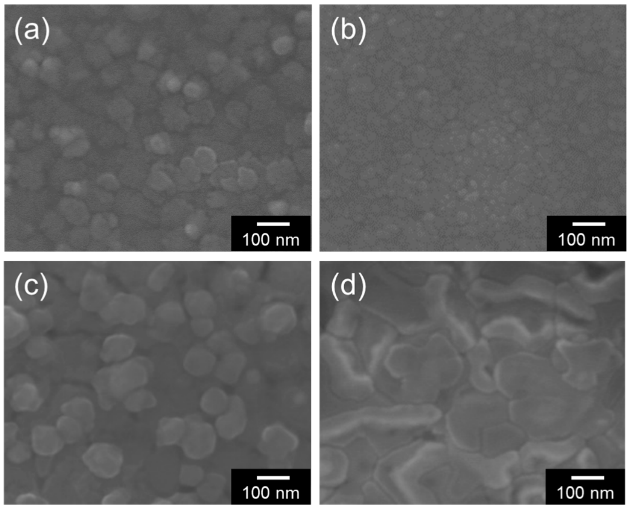

Figure S1. Surface FE-SEM image of the ZnVO layers grown at different temperatures: (a) 200 °C, (b) 300 °C, (c) 400 °C, and (d) 500 °C.

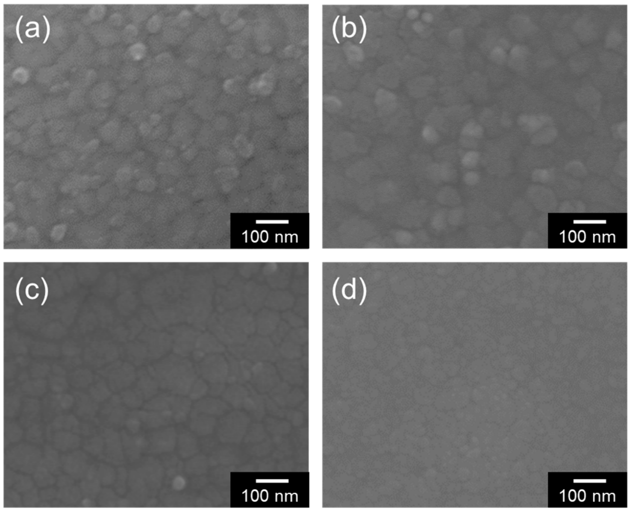

Figure S2. Surface FE-SEM image of the ZnVO layers grown under different gas flow conditions: (a) Ar : O_2 = 15 sccm : 10 sccm, (b) Ar : O_2 = 15 sccm : 15 sccm, (c) Ar : O_2 = 15 sccm : 18 sccm, and (d) Ar : O_2 = 15 sccm : 20 sccm.

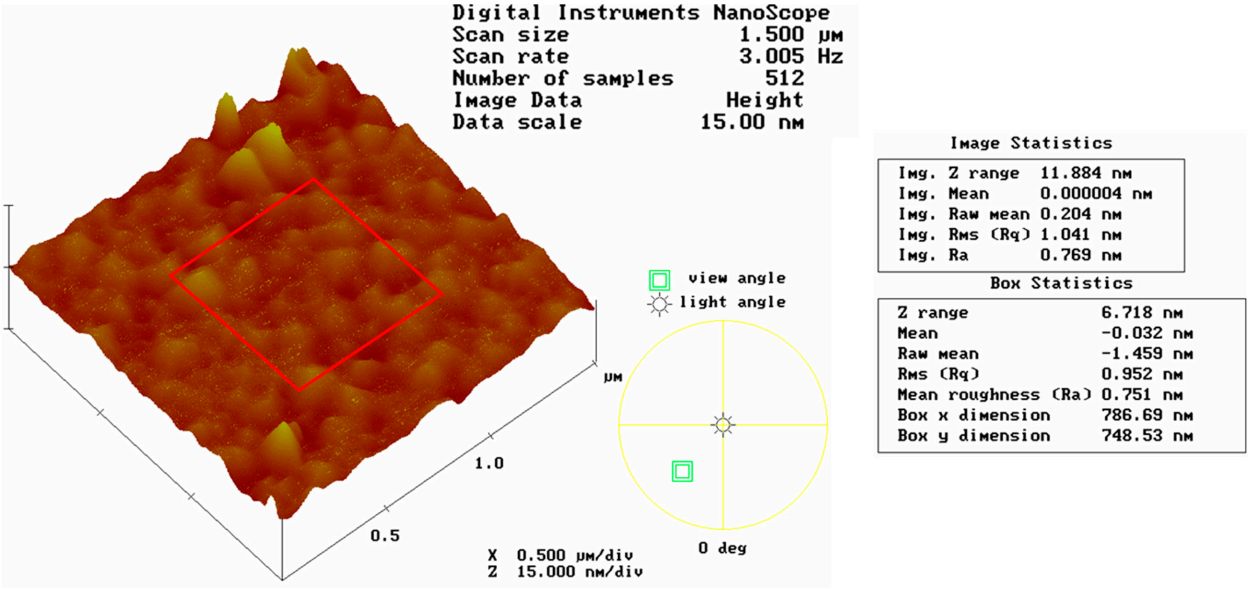

Figure S3. Surface AFM image of the ZnVO layer grown under at 300 °C in gas ambiance with Ar (10 sccm) and O_2 (20 sccm).

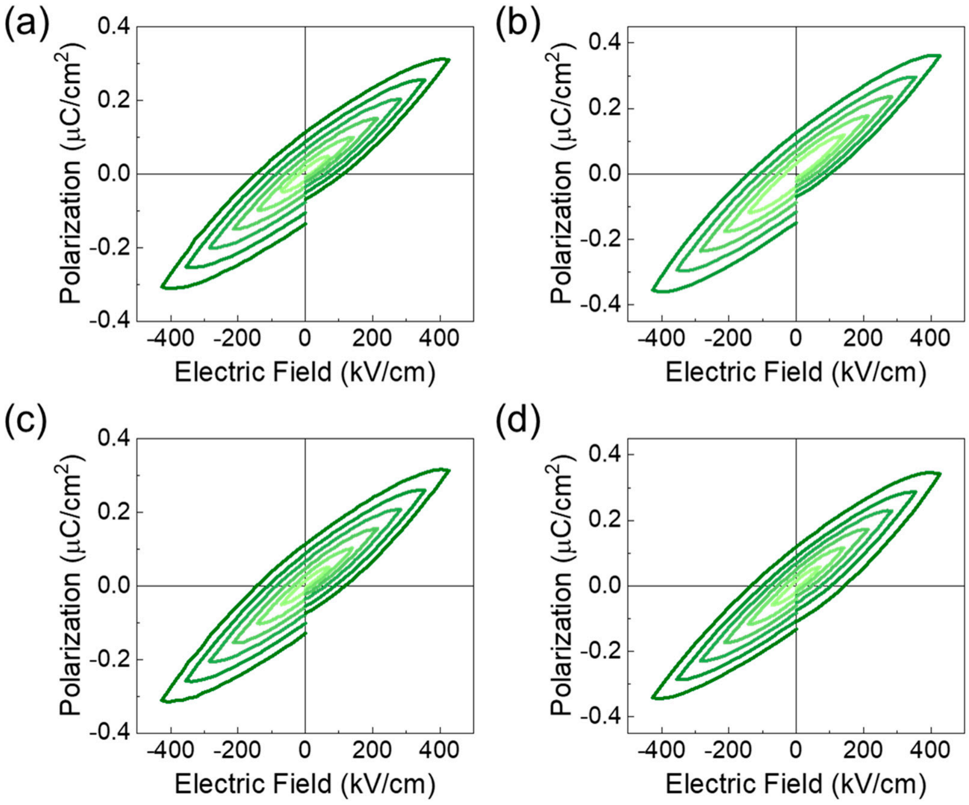

Figure S4. (a) – (d) P–E characteristics curves of the multiple ZnVO samples (obtained from different growth batches) grown at 300 °C in gas mixture of Ar (10 sccm) and O_2 (20 sccm).

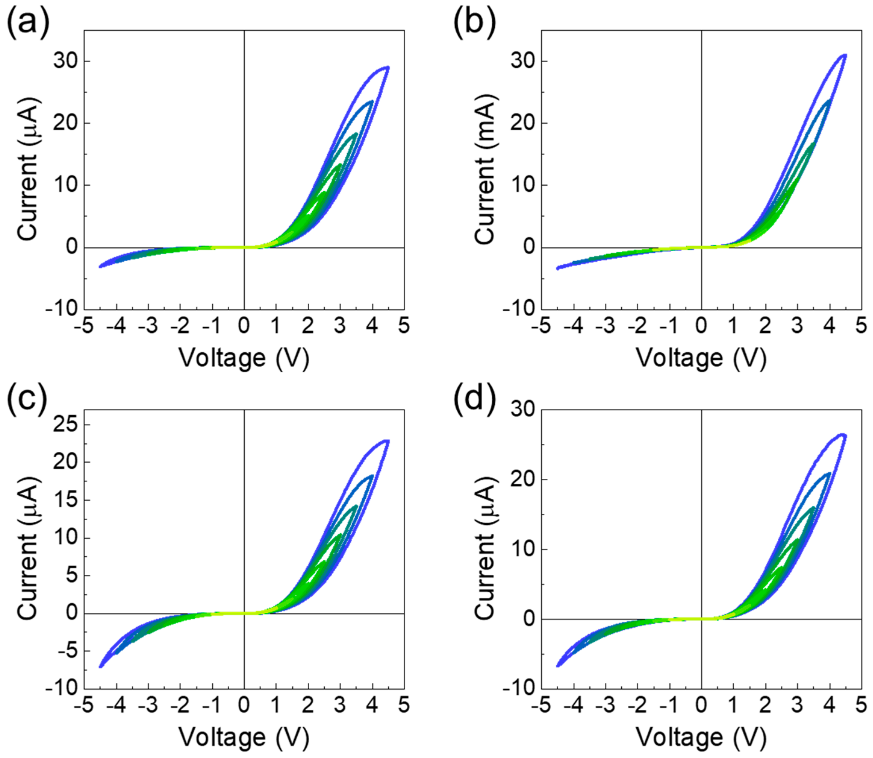

Figure S5. (a) – (d) I–V characteristics curves of the multiple Au/ZnVO/Pt FSJ devices that were fabricated using different ZnVO layers obtained from different growth batches. For all cases, the growth temperature and the gas flow rate were fixed at 300 °C and Ar : O_2 = 10 sccm : 20 sccm, respectively.

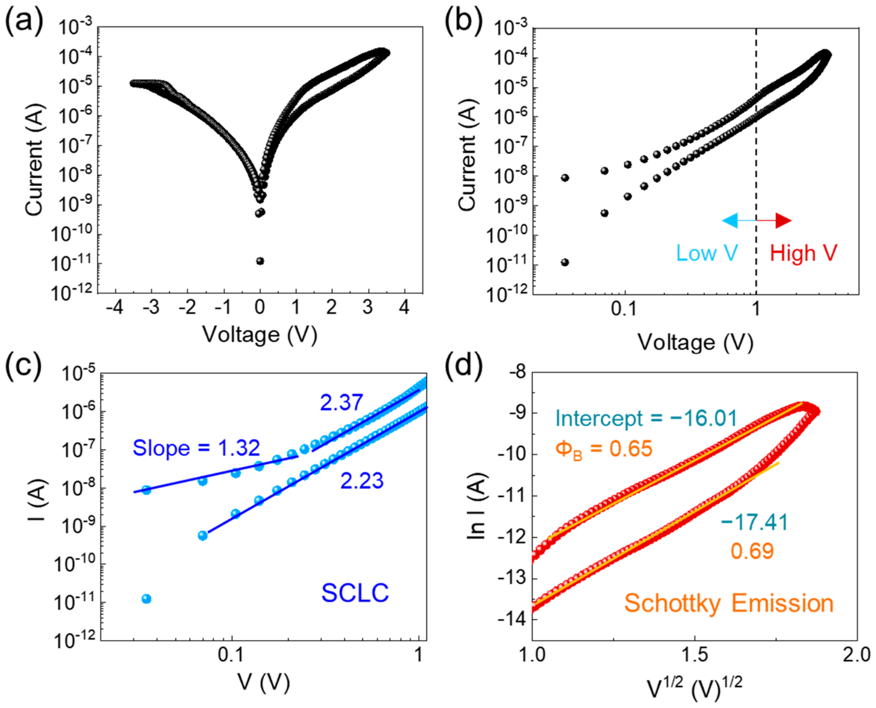

Figure S6. I–V characteristic curves of the Au/ZnVO/Pt FSJ device represented in (a) semi-logarithmic scale, (b) log-log scale, (c) space charge-limited conduction plot, and (d) Schottky plot.

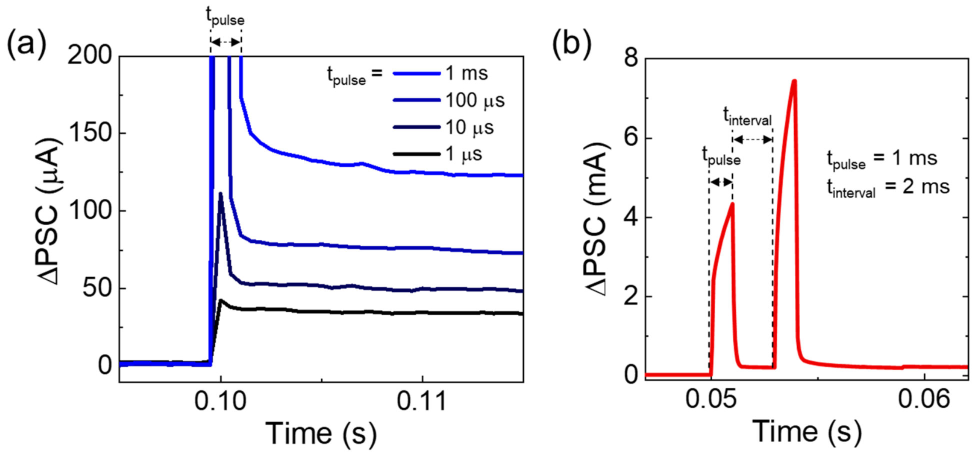

Figure S7. Transient characteristic curves for (a) EPSC and (d) PPF of the Au/ZnVO/Pt FSJ device.
